# Influence of Music upon Animals

**Published:** 1889-02

**Authors:** Moreau De Tours


### INFLUENCE OF MUSIC UPON ANIMALS.

TRANSLATED FROM JOURNAL D'HYGEINE, PARIS, FOR HALL'S JOURNAL.

One of the greatest proofs of the intelligence and extreme sensibility of animals, is their immoderate passion for music, and the aid which it often affords us in taming, subduing or moderating their ferocious character ; resisting their power or ardor, exciting their courage, developing their moral qualities and rendering them more generous. The horse redoubles his impetuosity at the thrilling sound of martial music, his eye glistens, his feet paw the earth : he is impatient of obedience to his master ; all a-foam he defies danger ; he achieves victory foaming, neighing, puffing, nimble, agile, impatient. Music which has redoubled his forces has doubled his endurance.

Moreover, we have the example of elephants, concerning which, much has been gathered in Paris and in London ; in the works of Pliny, of Sultonius, of Plutarch, etc., besides many others. In the public festivities of Rome we observe that he was trained to execute dances and military evolutions to the sound of music. In India horses fill an important place in the royal retinue, and have musicians assigned to their service. The camel, one of the animals earliest subjected to the service of man, learned to march to the cadences of song. We know the taste of the dog for music. Buffon relates that he has seen them leave their court yard to listen to a concert and return afterwards to their kennels. At the beginning of the Empire a certain dog attended regularly the parade at the Tuileries, and, taking his place between the knees of the musicians, marched with them, halted with them, and, after the review, disappeared to repeat the manœuvre the following day at the same hour. Sometimes this dog was not always so attentive, so silent, but howled if perchance, a musician blew a discordant note.

An angora cat about six months old, took great delight in sounding the notes of the piano. The moment her mistress left it open she would mount the instrument and pass and re-pass, without ceasing, along the keyboard for hours together, thus producing a jargon of discordant sounds, to her great delight.

In some parts of Germany and of Tyrol the hunters claim that they learn to attract the stag and the deer by playing upon the flute. We know with what pleasure birds, and above all canary birds, listen to airs which delight them.

It is also said that reptiles and insects are subdued by the same influences. The lizard, especially, has a great fondness for music. Upon hearing a voice or an instrument he shows at once by all his movements, that the sensation is agreeable : he turns sometimes upon his back, sometimes upon his paunch, or on his side. But he does not approve of all sorts of music ; he is quite particular in his tastes : a harsh voice or instrument displeases him. He likes a sweet voice, a tender air and a moderate movement.

Some travelers assure us that they have subdued the ferocity of the serpent by ringing a bell, by the sound of a flageolet, or by softly whistling. The same is said of many of the redoubtable vipers of Martinique. Chateaubriant, in the course of his journey to Upper Canada, gives an account of having seen a serpent that invaded his camp, made furious by bells, become calm at the sound of a flute and even follow after the musician.

Of all insects the spider is the most sensitive to music. It rapidly descends along its web and advances to the point whence proceeds the sound of instruments, there it will remain immovable even for hours, until the music ceases. One is familiar with the famous spider of Pellison.

It is unnecessary, however, to believe that this passion is general with animals of the same family : even those of the same species differ widely in disposition, some having a real antipathy for music which arouses their fury. Certain dogs become distracted and howl upon hearing the first sounds. It has the same effect upon them as the sound of cannon, causing them to slink away to their kennels and lie there quite unsociable and discontented, as if experiencing painful sensations, of which they preserve such disagreeable remembrances as to commence to bark should one touch a violin in their presence. There is an account of a dog that died of grief because he was forced for a long time to listen to music that was repulsive to him. A parallel example is found in the death of some other animals attributable to the same cause. Pierquin has had occasion to observe the effect of this antipathy upon a cat, even to bringing on convulsions during the whole period that it was made to hear selections from the operas sung with piano accompaniments, whilst another cat heard them with demonstrations of joy. Richard Mead informs us that owls have a very pronounced aversion to music.

So far, anatomy is unable to throw any light upon the antipathy of some, and the sympathy of others for music. We can only say it is a curious fact for consideration and solution.—DR. MOREAU DE TOURS.

